# Lasers in primary open angle glaucoma

**DOI:** 10.4103/0301-4738.73698

**Published:** 2011-01

**Authors:** Ramanjit Sihota

**Affiliations:** Glaucoma Research Facility and Clinical Services, Dr. Rajendra Prasad Centre for Ophthalmic Sciences, All India Institute of Medical Sciences, New Delhi, India

**Keywords:** Argon laser trabeculoplasty, laser-assisted deep sclerectomy, laser thermal scelrostomy, laser trabeculoplasty, selective laser trabeculoplasty

## Abstract

Lasers have been used in the treatment of primary open angle glaucoma (POAG) over the years, with the hope that they would eventually replace medical and surgical therapy. Laser trabeculoplasty (LT) is an application of argon, diode, or selective laser energy to the surface of the trabecular meshwork to increase the aqueous outflow. The mechanisms by which intraocular pressure (IOP) is lowered could be mechanical, biologic, or by division of adjacent cells. It is commonly used as an adjunct to medical therapy, but is contraindicated if the angle is obstructed, e.g., peripheral anterior synechia (PAS) or developmental glaucomas. About 75% of individuals will show a significant fall in IOP after argon laser trabeculoplasty (ALT), and the response is similar with selective laser trabeculoplasty (SLT). The effects of LT are not always long lasting, with about 10% of individuals showing a rise in IOP with every passing year. Laser thermal sclerostomy, *ab interno or externo*, is an alternative to other full-thickness filtration procedures. Longer wavelengths in the infrared range have water-absorptive characteristics that facilitate perforation of the sclera. These lasers can be used to avoid intraocular instrumentation and minimize conjunctival trauma.

Lasers have been used in the treatment of primary open angle glaucoma, POAG, over the years, with the hope that they would eventually replace medical and surgical therapy. So far, however, lasers have been found to lower the intraocular pressure (IOP) to a small extent and over a short term, in POAG. The purpose of this article is to provide an overview of the various laser procedures for the management of POAG.

## Laser Trabeculoplasty

Laser trabeculoplasty (LT) is the most common laser procedure used in the treatment of POAG.[1] It is an application of argon, diode, or selective laser energy to the surface of the trabecular meshwork (TM) to increase the aqueous outflow, as a treatment of glaucoma. The procedure has been used for a long time, but its position in the therapy of POAG, whether as primary therapy or after medical treatment, is found to be inadequate and is still to be determined. There are many advantages to this therapy, as patient compliance is not required, and its complications are few and minor.

### Mechanism of action

The mechanism by which laser applications influence IOP is not well studied; however, there are three major theories:[2–5]

1. Mechanical theory: This suggests that LT causes thermal changes in the trabecular beams, causing a contraction and shrinkage of trabecular beams at the site of application. This exerts a pull on surrounding beams, opening up the intertrabecular spaces.
2. Biologic theory: It is thought that laser energy causes tissue injury with a resultant cascade of events. Macrophages are attracted, and they alter the secreted extracellular matrix, allowing an increased aqueous outflow. Upregulation of interleukin I and tumor necrosis factor also upregulates metalloproteinase expression.
3. Cell division theory: Proponents of this theory propose that laser applications stimulate cell division in the anterior TM, providing pluripotent cells for the repopulation of lasered sites. Loss of endothelial cells over the trabecular beams causes the peripheral corneal endothelial cells to divide and slide over the lasered areas. These cells produce different extracellular matrix, enhancing the aqueous outflow.

In selective laser trabeculoplasty (SLT) the melanin in the TM is targeted with pulse duration, 3 ns, that is shorter than the thermal relaxation time of the tissues, thereby minimizing the thermal effects. The pulse duration or transmission of energy to the TM is about 1,000 times less in SLT. There is therefore no or little photocoagulation-induced thermal damage.[5]

### Indications

In POAG, the indications are as follows:

- when IOP remains above “target” IOP despite maximally tolerated medical therapy
- when patients find it difficult to administer drops or are noncompliant
- pseudoexfoliation glaucoma
- when surgery needs to be deferred due to a patient’s systemic status.

In secondary glaucomas, the indications are as follows:

- pseudophakic glaucoma
- glaucoma with pseudophakia.

The contraindications are as follows:

- Narrow angles
- Presence of peripheral anterior synechiae
- Uveitis
- Advanced glaucoma
- Developmental glaucomas.

### Methodology

Various antireflective lenses may be used for LT, the clinical gonioscope, a four-mirror lens, or the Ritch trabeculoplasty lens which has two mirrors at 59°, permitting a view of the inferior angle, and two others inclined at 64°, for viewing the superior angle. Two of these mirrors have a planoconvex lens overlying them, to produce ×1.4 magnification, reducing the spot size and increasing the laser power by a factor of 2. A Latina lens, made of quartz is used for SLT.

The eye is pretreated with a drop of brimonidine or a beta blocker, if not contraindicated, just before or immediately after the procedure.

In argon laser trabeculoplasty (ALT), a spot size of 50 μm, 0.1-s duration, is applied to the junction of the anterior pigmented and posterior nonpigmented TM, with the power titrated to achieve blanching. The spot should be well focused and circular for best results. The power required depends on the pigmentation of the TM, and in Indian eyes is generally around 800 mW. Power adjustments may be required in different quadrants of the same eye. Approximately 100 laser spots are equally spaced over 360° of the angle, or 50 over 180°.

SLT utilizes a spot size of 400 μm and starts with a power of around 0.8–1 mJ for lightly pigmented TM. The power can be titrated till it causes champagne bubbles to form. Confluent spots are applied for best results. In more heavily pigmented TM, around 0.6 mJ may be used. A total of 360° of the TM is lasered, except in an eye with a heavily pigmented TM, when 180° is done first, and the remaining 180° later, if needed.

The spot size of ALT is 50 μm, compared to 400 μm with SLT. The size determines the energy delivered per unit area, so that ALT uses 40–70 mJ per pulse versus 0.6–1.2 mJ by SLT. In ALT, the pulse duration is 0.1 s as compared to 3 ns in SLT [Table 1].

**Table 1:** Comparison of ALT and SLT techniques

	Argon laser trabeculoplasty	Selective laser trabeculoplasty
Spot size	50 μm	400 μm
No. of spots	50 spots	50 spots
Energy used	500 mW	<1% of ALT
Fluence	40,000 mJ/mm^2^	>0.00015% of ALT
Exposure time (s)	0.1	0.0000000003
Effect	Thermal damage	No thermal damage

### Efficacy

This can only be assessed after 6 weeks, at about 3 months, when all cellular and biologic changes have taken effect. About 75% of individuals will show a significant fall in IOP after ALT, and the response is similar with SLT. The effects of LT are not always long lasting, with about 10% of individuals showing a rise in IOP with every passing year.[6–9] Repeat ALT does not give more than 2–3 mmHg of a fall in IOP, whereas SLT has been shown to be more effective as a repeat procedure, in about 50% of eyes, both after ALT and SLT. The glaucoma laser treatment trial showed that 7 years after ALT, laser-treated eyes had a lower IOP, better fields, and better optic nerve head parameters, than eyes treated with medication. Twenty-five percent of ALT eyes did not require any other medication.[6] Five-year follow-up of SLT has shown an approximate 20% reduction in IOP, in 60–70% of eyes.[10–18] Damji *et al*. have shown similar efficacy for ALT and SLT over time.[14,18] Factors related to the success of LT were a higher baseline IOP and more pigmented eyes [Table 2].

**Table 2:** Long-term comparisons of argon laser trabeculoplasty and selective laser trabeculoplasty

	SLT sample	ALT sample	SLT IOP drop	ALT IOP drop	Follow up
Damji *et al*.	18	18	4.8 mmHg	4.7 mmHg	6 months
Popiela *et al*.	27	27	2.85 mmHg	2.6 mmHg	3 months
Bovell *et al*.	64	68	1yr:6.5 mmHg 2yr: 4.5 mmHg	5.7 mmHg 5.9 mmHg	2 yrs
Damji *et al*.	36	33	6.5 mmHg	6 mmHg	3yrs
Hong *et al*.	20	25	6.3 mmHg	3.7 mmHg	3 months
Martinez-de la Casa *et al*.	20	20	22.2%	19.5%	6 months

### Complications

A potential complication is a temporary increase in IOP, which is treated with drugs. The effects of LT are not always long lasting:

1. IOP can spike in 3–5% of eyes, commonly occurring 1–4 h after the procedure.
2. Peripheral goniosynechiae can be seen after both ALT and SLT.
3. A chronic rise in IOP may be seen in a few eyes with both ALT and SLT.
4. Corneal haze and scarring have been reported after SLT.
5. Iritis and even choroidal effusion have been reported after SLT.

Failure of trabeculoplasty may be a result of

1. argon laser burns in TM causing thermal damage to collateral tissues
2. repair of injured TM with scarring (increase collagen tissue within TM)
3. migration of corneal endothelial cells to the TM
4. filling up of intertrabecular spaces with collagen and corneal endothelial cells
5. increased obstruction to aqueous outflow.

Laser trabeculoplasty as ALT and SLT, or by other lasers,[19] is a safe procedure for a similarly moderate lowering of IOP. The question about collateral damage during the procedure is still to be definitively answered.

## Laser-Based Filtration Procedures

Lasers are being investigated to replace or augment traditional glaucoma filtration surgery. Many procedures have been tried experimentally; however, there are a few in clinical trials currently.

### Laser thermal sclerostomy *ab interno* or *externo*

Laser thermal sclerostomy, *ab interno* or *externo*, is an alternative to other full-thickness filtration procedures. Longer wavelengths in the infrared range have water-absorptive characteristics that facilitate perforation of the sclera. These lasers can be used to avoid intraocular instrumentation and minimize conjunctival trauma.

A thulium, holmium, chromium-doped YAG crystal laser[20] has been evaluated to create thermal *ab externo* sclerostomies. The near infrared laser, 2100 nm, is a long-pulsed, compact, self-contained, solid-state laser operating in the near-infrared. Energy was delivered via a specially designed 22-gauge optic probe that emits energy at a right angle to the long axis of the fiber. This can therefore be passed subconjunctivally to the limbus. Pulse energies of 80–120 mJ were used. Total energy levels to produce full-thickness sclerostomies ranged from 1.4 to 7.2 J. Subconjunctival 5-fluorouracil injections were administered. Twenty-five percent of cases failed within the initial 6 months, and 5% additionally by 12 months.

Full-thickness *ab externo* sclerotomies have been shown to produce early hypotony, and a high rate of failure over time.

An *ab interno* sclerotomy has been advocated to prevent scarring of the conjunctiva. A continuous wave neodymium YAG laser has been tried on rabbits, creating two sclerostomies *ab interno* on one eye in rabbits, using a 200-μm diameter quartz optical fiber. A viscoelastic substance is injected subconjunctivally in the area of the proposed bleb. The probe is passed across the anterior chamber, to the TM, where the sclerostomy is made. Well-defined filtering blebs were seen immediately postoperation, but after the fifth day, the conjunctival blebs had disappeared. After thermal stress had faded, the collagen appeared to undergo a process of repolymerization. By day 10, the lumen had become occluded by a loose meshwork of phagocytes, fibroblasts, and proliferating capillaries. These new vessels and the loose nature of the canal-occluding framework and of the surrounding regenerating collagenous tissue could have further permitted percolation and transport of aqueous humor, since IOP remained low, despite the disappearance of filtering blebs. Eighty percent of full-thickness fistulas remained patent, maintaining significant IOP reduction; the others were blocked by the iris. A pilot therapeutic trial was conducted in pseudophakic patients with advanced open angle glaucoma. Six full-thickness sclerostomies and three guarded sclerostomies were created in nine patients by 193-nm excimer laser ablation. After 6 months’ follow-up, IOP was controlled in eight of the nine patients. Early postoperative complications included hyphema, temporary fibrinous sclerostomy occlusion, profound early hypotony, and suprachoroidal hemorrhage in one case. Conjunctival laser wounds were self-sealing. Small-bore laser sclerostomy procedures are functionally equivalent to conventional full-thickness procedures, producing early postoperative hypotony, with an increased risk of suprachoroidal hemorrhage in association with this. Iris incarceration is the most common problem associated with laser sclerostomies. Overdrainage, hypotony, and cataract formation are also commonly seen. Laser sclerostomy retains all the risks of a free filtering procedure when it works, and also has a high risk of failure. It is currently still being evaluated.

### Laser deep sclerectomy

Carbon dioxide (CO_2_) lasers have the inherent property of losing effectiveness when in contact with liquid; the full penetration of the sclera by mistake is almost impossible. They have therefore been studied for use in nonpenetrating deep sclerectomies. The CO_2_ laser ablates the sclera to an optimal thickness that allows percolation of the intraocular fluid, as the CO_2_ laser essentially stops ablating as soon as it comes in contact with the percolating aqueous.[21]

#### Erbium YAG-laser-assisted deep sclerectomy[22,23]

Klink *et al*.[22] dissected the deep corneoscleral lamella in deep sclerectomy with a pulsed erbium YAG laser, with energy 40–100 mJ and frequency 5–10 Hz. Schlemm’s canal was unroofed and the lamella thinned until the aqueous percolated continuously through the membrane. The complete success rates were 83.3% at 3 months and 50% at 12 months. Qualified success was 91.7% at 3 months, 92.9% at 12 months, and 78.6% at 50.5 months. The number of glaucoma medications was reduced from 3.07 ± 0.92 preoperatively to 1.14 ± 1.41 at 50.5 months. A single case of anterior-chamber penetration, requiring iridectomy, was the only intraoperative complication.

#### The use of erbium

YAG laser for performing a deep sclerectomy procedure seems to be a safe and relatively effective alternative to the conventional surgical procedure.

In summary, lasers have been used successfully over the years in POAG for adjunctive IOP lowering by a trabeculoplasty, while other procedures are still being developed.
